# Combustion-derived nanoparticles: A review of their toxicology following inhalation exposure

**DOI:** 10.1186/1743-8977-2-10

**Published:** 2005-10-21

**Authors:** Ken Donaldson, Lang Tran, Luis Albert Jimenez, Rodger Duffin, David E Newby, Nicholas Mills, William MacNee, Vicki Stone

**Affiliations:** 1 ELEGI Colt Laboratory, Queens Medical Research Institute, University of Edinburgh, 47 Little France Crescent, Edinburgh EH16 4TJ, UK; 2 Institute of Occupational Medicine, Research Park North, Riccarton, Edinburgh EH14 4AP, UK; 3 Cardiovascular Research, Division of Medical and Radiological Sciences, The University of Edinburgh, Chancellor's Building, 49 Little France Crescent, Edinburgh EH16 4SU, UK; 4 Napier University, School of Life Sciences, 10 Colinton Rd, Edinburgh EH10 5DT, UK

## Abstract

This review considers the molecular toxicology of combustion-derived nanoparticles (CDNP) following inhalation exposure. CDNP originate from a number of sources and in this review we consider diesel soot, welding fume, carbon black and coal fly ash. A substantial literature demonstrates that these pose a hazard to the lungs through their potential to cause oxidative stress, inflammation and cancer; they also have the potential to redistribute to other organs following pulmonary deposition. These different CDNP show considerable heterogeneity in composition and solubility, meaning that oxidative stress may originate from different components depending on the particle under consideration. Key CDNP-associated properties of large surface area and the presence of metals and organics all have the potential to produce oxidative stress. CDNP may also exert genotoxic effects, depending on their composition. CDNP and their components also have the potential to translocate to the brain and also the blood, and thereby reach other targets such as the cardiovascular system, spleen and liver. CDNP therefore can be seen as a group of particulate toxins unified by a common mechanism of injury and properties of translocation which have the potential to mediate a range of adverse effects in the lungs and other organs and warrant further research.

## Introduction

Particulate matter (PM) is a complex mixture of different particle types, much of which are unlikely to cause any adverse effects and so the hypothesis has arisen that a sub-component(s) of PM drives the adverse effects. Much research has been, and is being, undertaken to test various hypotheses regarding which of the components in fact drives adverse effects. Combustion has been recognised as a potential source of harmful particles as well as gaseous pollutants [1,2]. Epidemiological studies do not readily allow associations of adverse effects with sub-components of PM, dependent as they usually are on mass measures of PM. However several epidemiological studies have been able to identify combustion-derived particles as an important component in driving adverse effects of PM [3-7]. Toxicology can more readily study the components of PM and there has been considerable amount of research demonstrating the toxicity of combustion-derived particles such as diesel soot [8,9], welding fume [10], carbon black [11] and nanoparticles coal fly-ash [12]. The workplace is also a site of exposure to combustion-derived nanoparticles as in the case of welding fume and in the manufacture of carbon black. We focus here on the toxicology of combustion-derived nanoparticles (CDNP) produced in a range of situations because reports on their mechanisms of toxicity suggest similarities.

**Figure 1:**
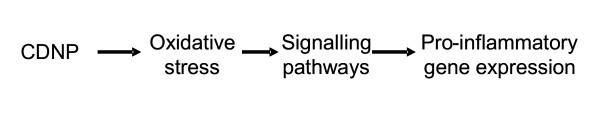


CDNP present a diverse group of materials which gain commonality because of their origin in combustion processes and their demonstrated toxicity in various models. We have attempted to be systematic and have described the CDNP in terms of their physicochemistry then their adverse effects and finally their molecular toxicology; however there are gaps of various sizes in the available information and so we fall short of a truly systematic approach. We feel that this review is timely because the molecular toxicology of these materials is becoming better understood and the final common pathways of oxidative stress-mediated inflammation are now considered to underlie the effects of a range of CDNP. This is outlined in Figure 1 where the link between oxidative stress and inflammation is shown. In addition we review the evidence that CDNP and their components can migrate, from their site of deposition in the lungs, to other target organs.

## Combustion-derived nanoparticles

Nanoparticles are defined as primary particles with at least one dimension < 100 nm, while ultrafine particles are defined as particles < 100 nm in all dimensions (W Kreyling, personal communication) and are commonly produced by combustion processes [1,2]. Like other nanoparticles, CDNP agglomerate readily and move into the accumulation mode which decreases the particle number but probably leaves the surface area dose unaffected. NP have the ability to cause inflammation and also, in the case of insoluble CDNP, have potential to escape from the site of deposition in the lungs and translocate to the blood and to other target organs [13]. The exemplar CDNP discussed here (Table 1) include welding fume and nanoparticulate carbon black, which are both occupational hazards, coal fly-ash which is an environmental hazard and diesel soot which is both an environmental and an occupational hazard. CDNP are primary in the sense that they arise directly from the combustion process, although their chemistry may change with aging as the particles undergo chemical interactions with components of the ambient air pollution cloud. The process of burning concentrates metals, due to combustion and hence degradation of the organic fraction to a degree that is dependent on the efficiency of the combustion. At the same time pyrolysis chemistry generates other complex organic molecules, some of which may persist along with elemental carbon nanoparticles. Nanoparticles also form from atmospheric chemistry e.g. sulphate and nitrate nanoparticles but these will not be discussed here as they are not derived directly from combustion. The combustion materials and the mode of combustion will ultimately determine the characteristics of the CDNP, including chemical composition, particle size and particle solubility. The large surface area of CDNP presents maximal opportunity for dissolution of soluble species from the surface of the insoluble core. For insoluble NP, the large surface area provides a surface on which catalytic chemistry can occur that favours the formation of free radicals. These free radicals are responsible for driving oxidative stress, the underlying mechanism that promotes an inflammatory response to CDNP. For a range of low toxicity, low solubility particle types the surface area alone is the driver for lung inflammation following instillation in rats [14]. CDNP may be soluble and release transition metals or organics as their primary pro-inflammatory mechanism. Both transition metals and organics can undergo complex cyclical chemical reactions in the milieu of the lungs that lead to the production of free radicals such as superoxide anion or hydroxyl radical [15-17]. By contrast low toxicity insoluble particles cause inflammation because of their surfaces; some types of CDNP have both soluble components and an insoluble core.

**Table 1:** Characteristics of the CDNP considered in this review

**CDNP**	**Origin**	**Reported health effects**	
		
		**animals**	**humans**
Diesel exhaust particles	Combustion of diesel oil	Inflammation, fibrosis, cancer,	Inflammation, cancer?
Welding fume	Welding processes	Inflammation; translocation of metals to the brain	Metal fume fever, fibrosis, cancer, bronchitis
Fly-ash	Combustion of coal or oil	inflammation	no data available
NP Carbon black	Combustion of heavy fuel oil	Inflammation, lung cancer; translocation of particles to the brain	no data available

As detailed in this review, exposure to CDNP of various types is associated with a range of adverse health effects including fibrosis, chronic inflammatory lung disease, metal fume fever and cancer. These endpoints are found across a number of exposure conditions and to different kinds of CDNP but are not unique for CDNP.

This paper examines the evidence for harmful effects of CDNP and puts these in the context of a unifying hypothesis based on observations that the generic ability of CDNPs to cause inflammation is via oxidative stress and activation of redox-sensitive transcription factors that can lead to the adverse health effects listed below. The ability of CDNP and their associated metals to translocate to the blood and the brain are also discussed. These unusual toxic properties unite these materials and suggest that they can usefully be seen as a group of particulate lung toxins that act through similar pathways.

## Health effects associated with exposure to CDNP

CDNP are generated in a number of scenarios including internal combustion engines, large scale burning of coal for power generation and in industrial processes where they often could be produced along with larger particles. The CDNP considered here are described in Table 1 along with their salient effects in humans and animals.

### Diesel exhaust particulate

Both petrol and diesel fuels undergo combustion in automobile engines and give rise to CDNP [18,19] but diesel produces more particles per unit fuel than petrol and is by far the most-studied of the two regarding adverse health effects; therefore diesel CDNP are discussed here. Diesel fuel is a middle distillate of petroleum which contains paraffins, alkenes and aromatics[20]. On combustion in automobile engines it produces low solubility carbon-centred nanoparticles with complex chemical and physical structure, containing sulphates and an organic fraction comprising unburnt fuel, lubricating oil and polycyclic aromatic hydrocarbons along with a range of other chemicals, which can condense on the particles [20,21]. Singlet Diesel nanoparticles are 5–20 nm but readily form complexes chains and aggregates of 60 to 100 nm and larger [22]. Diesel exhaust particles (DEP) are usually the most common CDNP in urban environmental air and in environmental particulate air pollution (PM_10_) in conurbations generally; they also occur in an occupational setting. In the ambient environment the concentration of DEP in PM_10 _is likely to range from 5–30 μg/m^3 ^while in occupational settings levels up to 1000 μg/m^3 ^have been experienced [20]. The adverse health effects of exposure to DEP have been extensively studied epidemiologically, in animals and in cells. Epidemiological studies have been reviewed and show that there is a strong link between occupational exposure to diesel soot and lung cancer [20]. Animal studies generally support these findings and demonstrate that exposure to DEP and other nanoparticulate forms of carbon are carcinogenic [23] but these findings are complicated by the issue of rat lung overload [24]. Rat lung overload is a condition when very high lung surface area burden [25] of low toxicity, low soluble particles leads to failure of clearance, rapid accumulation of dose with concomitant inflammation and proliferation, which culminates in fibrosis and cancer. Humans are unlikely to experience overload levels of diesel soot, even in occupational settings, and there is a question over whether overload can occur at all in humans. It is therefore unlikely that cancer associated with DEP-exposure in humans results from mechanism similar to rat lung overload. Exposure to DEP has also been shown to be highly inflammatory in rats and mice in non-overload conditions [26-28] and to induce pro inflammatory effects on cells *in vitro *[29-31]. The well-documented link between inflammation and lung cancer [32-34] supports the idea that diesel exhaust may indeed be carcinogenic via an inflammatory pathway (see below).

Many studies have demonstrated profound adjuvant effects of diesel particles on development and intensity of allergic responses and these effects are mediated by direct effects of DEP on a wide range of cell types involved in allergy [35]. These effects could also be mediated indirectly through inflammation and oxidative stress [35]. The inflammatory effects of DEP appear to be driven by the particulate component i.e. the surface area effect [23] although the organic [36] and metal components [37] also appear to play a role in oxidative and pro-inflammatory effects and thereby affect pathogenicity.

### Welding fume

Welding is an industrial technique that involves the joining of metal pieces using a filler metal. The filler metal is produced from an electrode wire that is consumed during the welding fusion process. High temperatures are involved, generating a welding fume as well as radiation, noise and gases [38] but we focus her on the fume particles. The vaporized metal produced by the heat of the welding process oxidises to produce a fume containing particles of metal oxide such as aluminium, cadmium, chromium, and copper [38], many of which are water soluble. The exact composition of the welding fume is determined by the metals involved in the weld and the composition of the electrode. Welding fume particles are comprised of a large proportion of nanoparticles [10].

Exposure to welding fume has been associated with both pulmonary and systemic health endpoints reviewed in [38]. These include decreases in pulmonary function, increased airway responsiveness, bronchitis, fibrosis, lung cancer and increased incidence of respiratory infection; in addition to these pulmonary effects metal fume fever is frequently observed in welders [38,39]. This systemic condition is considered to be caused by inhalation of zinc oxide fumes and it is characterised by acute onset of a flu-like illness accompanied by a dry cough, dyspnea, muscle aches, headaches and fever [40]. Metal fume fever is usually experienced in the first periods of exposure and on Mondays, with the symptoms declining as the working week progresses. Welding fume has been studied in both animals and in cells in culture, and in both it produces marked pro-inflammatory effects [10,41,42]. These effects are driven largely by the transition metals [10,42,43] which undergo redox-cycling resulting in oxidative stress.

### Nanoparticulate carbon black

Carbon black (CB) is a low solubility particle produced industrially from incomplete thermal decomposition of hydrocarbons [44] in which the process is controlled to achieve pre-defined and reproducible particle sizes and properties suitable for a diverse range of industrial applications. Unlike the other CDNP described here NPCB is not accidentally produced, and is an industrial product but it clearly classifies as a CDNP. In thermal-oxidative processes such as the furnace black process, various types of hydrocarbon are sprayed into a natural gas-fired furnace and quenched with water to prevent complete burning [45]. The carbon black particles so-formed are complex, with a degenerated graphitic crystallite structure and high power electron micrographs clearly show irregular layered graphitic plates. [44]. The structure of carbon black is described as nodules, the roughly spherical primary structural elements, aggregates which comprise fused, connected particles and agglomerates, which are undispersed clusters of aggregates. CB has been studied extensively as to its toxicology, especially as an example of a low toxicity, low solubility particle not complicated by harmful levels of toxicologically-relevant organics or metals [23]. In long-term animal studies CB was found to be a carcinogen although rat lung overload very likely plays a role in this affect [46].

The smallest nodule-or particle-sized CB comprises primary particles in the low tens of nanonetres size range. CB with smallest primary particle sizes produces the highest optical density (jetness) compared to larger particle sizes, placing this material in demand for colouring enamels, acrylics and plastics, as well as inks and paints [44]. This nanoparticulate CB (NPCB), as it has come to be known, comprises a portion of the overall CB industry. NPCB was able to cause detectable but low level pro-inflammatory effects in rats following 7 hours inhalation exposure [47] and also following instillation [11] and this appears to be a consequence of the high surface area area per unit mass [14]. In cell studies NPCB has been shown to cause oxidative stress, pro-inflammatory gene transcription [48] stimulation of phagocytosis at low doses and inhibition at higher doses [49]. In studies of the health status of individuals working in the carbon black industry there is evidence of abnormalities in chest radiographs and respiratory morbidity, but equivocal findings on lung cancer [50-52]. However, none of these studies analysed a worker population exposed solely to NPCB. NPCB has been used quite extensively in particle toxicology as a model particle and nanoparticles, so there is a considerable existing database on its toxicity in vitro and in vivo [53].

### Coal fly ash

Pulverised coal combustion is a commonly-used and efficient method of coal burning in power stations. In pulverized coal power stations the pulverised coal is blown into the furnace and burned off producing a fly ash emission. This particulate emission is controlled by number or methodologies including electrostatic precipitators, filters scrubbers and mechanical collectors [12]. However, these control measures are not 100% effective and some particles are released into the environment.

Toxicology studies have in general examined unfractionated pulverised coal fly ash (CFA) and these have shown generally low toxicity [54], however bioavailable iron has been reported to underlie an ability to generate oxidative stress [55,56]. Coal fly ash-exposed rats have been shown to exhibit increased susceptibility to infection [57], while a specially-prepared cloud of ultrafine (nanoparticulate) coal fly ash induced adverse effects on guinea pig lung function [58]. A recent study systematically examined the effect of fractionated coal fly ash in pulmonary inflammation and a nanoparticle fraction was available [12]. This study showed greatly enhanced potency of the nanoparticulate fraction compared to the fine and coarse fractions, as seen by enhanced ability to cause lung inflammation and kill macrophages in culture. The nanoparticle fraction was not especially enriched for toxic metals and the increased toxicity of this fraction may be a result of the high surface area, allowing redox reactions to take place.

## Molecular toxicology mechanisms driving the inflammatory effects of CDNP in lungs

Based on published studies we hypothesise that CDNP have their effects through common pathways that produce inflammation and that oxidative stress is the lead effect driving the adverse health effects. Table 1 shows the CDNP studied in this paper along with a description of their origin and reported health effects in animals and humans.

**Figure 2:**
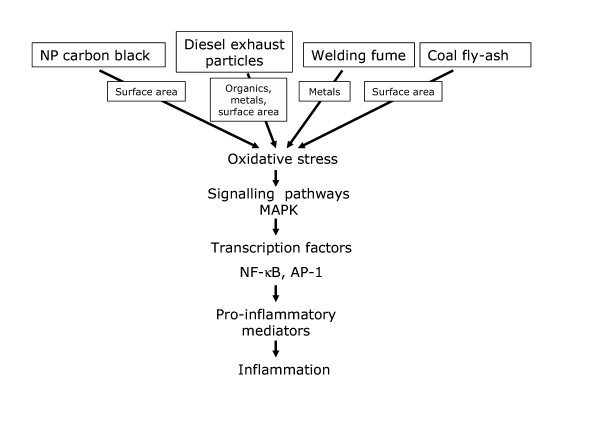


There is considerable mechanistic data describing the molecular events flowing from the deposition in the lungs of the different CDNP under discussion here and this is outlined in Figure 2. Figure 2 shows that different components of different CDNP can cause oxidative stress that acts through well-documented redox-sensitive pathways, such as MAPK and NF-κB, to cause inflammation. Although the components that mediate these effects differ greatly between the different CDNP, there is commonality through their ability to cause oxidative stress and inflammation.

### Diesel exhaust particles

As described above DEP causes inflammation in rat lungs [28,59] and in human lungs [60] following short-term, high level exposure. Evidence of the oxidative properties of DEP *in vivo *is shown by increased level of 8 OH dG, the oxidative adduct of hydroxyl radical, in the lungs of rats following exposure and in cells in culture treated with DEP [61,62]. DEP causes oxidative stress in a number of models *in vitro *such as oxidation of low density lipoprotein (LDL) [63] and in exposed epithelial cells [9,64]. The component responsible for the oxidative stress and subsequent pro-inflammatory signaling is principally the organic fraction [9,30,64,65], although transition metals may also be involved [37,66]. The organic fraction either contains, or can be metabolized to, species such as quinones that can redox cycle in cells to generate reactive oxygen species [17]

Activation of signaling pathways for pro-inflammatory gene expression is seen in a number of studies using DEP; these include MAPK activation [30,67-69] and NF-κB activation [30,70]. As would be anticipated, activation of these pathways culminates in transcription of a number of pro-inflammatory genes such as IL-8 in epithelial cells treated *in vitro *[71] and in human lungs exposed by inhalation [72]. TNFα has been reported to be increased in macrophages exposed to DEP *in vitro *[73] and IL-6 is released by primed human bronchial epithelial cells exposed to DEP [74]. Increased expression of the GM-CSF gene is reported in human epithelial cells exposed to DEP; in humans exposed to short-term high levels of DEP similar to those encountered in a busy garage, bronchial biopsies showed increased GROα and RANTES expression in the bronchial wall [75].

### Nanoparticulate Carbon black (NPCB)

As described previously, at high exposures carbon black causes overload tumours in rats [27,46]. NPCB causes inflammation and the onset of rat lung overload tumours at a lower lung mass burden than larger, respirable CB [76]. This reflects the reliance of rat lung overload on the particle surface area burden [25], which is much greater for a given mass of NPCB than the same mass of non-NP respirable CB. Even at low lung burden NPCB showed evidence of mild pro-inflammatory effects whilst respirable CB did not [47]. Similar greater inflammogenicity of NPCB than respirable CB has been described with instillation models, [11,77].

Reactive oxygen species production has been measured with NPCB using *in vitro *cell-free systems [78,79] and oxidative stress has been demonstrated in exposed cells [48,80]. The chemical basis of the ability of NPCB to cause oxidative stress [78] is unknown, but unlike highly soluble welding fume, ROS production is not related to metal or any other soluble component [77]. In a cell free system the NPCB particles and similarly polystyrene NP, induce ROS production [81], suggesting that the surface reactivity is sufficient. However, in cells this ability may also be related to increased influx of extracellular Ca^++ ^ions seen with NPCB [82]. Oxidative stress raises intracellular calcium by increasing release of Ca^2+ ^from the endoplasmic reticulum, by enhancing ingress of Ca^2+ ^through the plasma membrane calcium channels and through inhibition of Ca^2+ ^transport out through the ATPase pumps in the plasma membrane [83]. Oxidative stress caused by NPCB is translated into activation of NF-κB and IL-8 gene expression in epithelial cells *in vitro *[48], while both oxidative stress and calcium are implicated in activation of AP-1 and TNFα production in macrophages [84]. A recent study reports that NPCB causes oxidative stress-mediated proliferation of airway epithelium, involving the Epidermal Growth Factor Receptor and the ERK cascade [85].

### Welding fume

Exposure to welding fume nanoparticulate in humans is associated with inflammatory cytokine increases in the bronchoalveolar lavage (BAL) [86-88] and systemic oxidative stress [43]. The ability of welding fume to generate free radical is abundantly clear, even in a cell-free environment with only H_2_O_2 _acting as a reductant [42]. Rats exposed to welding fume show marked pulmonary inflammatory responses [42,89,90] and lipid peroxidation indicative of oxidative stress [42]. In a comprehensive study of the molecular signaling pathways leading to inflammation with welding fume, McNeilly et al demonstrated that the pro-inflammatory effects of welding fume *in vitro *[10] and *in vivo *[91] were entirely driven by oxidative stress arising from the soluble transition metal component. Epithelial cells treated with welding fume or the soluble transition metals from them showed oxidative stress leading to MAPK-dependent (manuscript in preparation) NF-κB and AP-1 activation leading to IL-8 gene transcription [10]. For welding fume nanoparticles, therefore, the soluble transition metals appear to be the primary mechanism of oxidative stress and inflammation.

### Coal fly ash

In the past coal fly ash has been shown to have relatively low toxicity, for example lower than coal or quartz [54]. Recently there has been increasing interest in the ability of CFA to release bioavailable iron which can redox cycle to produce oxidants [55,92]. One study showed that the ability of a CFA standard to induce IL-8 release from epithelial cells was dependent on size, with the smallest size fraction (<1 μm) containing the most IL-8-stimulating activity [92]; this was due to the fact that the bioavailable iron was concentrated in this fraction. There was no attempt in this study to collect a nanoparticle fraction. In a study especially relevant to our review of CDNP, Gilmour et al [12] demonstrated that the nanoparticulate fraction of sub-bituminous coal was much more potent than any other fraction in causing lung inflammation and cytotoxicity *in vitro*, when compared on a mass basis. This was not obviously linked to enrichment of Fe or any other toxic metals in the nanoparticulate fraction. Electron microscopic examination of the nanoparticulate fraction of coal fly-ashes from bituminous and low-rank coals showed abundant discrete crystalline particles rich in Fe, Ti, and Al crystalline phases down to 10 nm in size whilst low-rank samples contained considerable amounts of alkaline-earth element aggregates in the form of phosphates, silicates, and sulfates and mixed species. Importantly, all coal fly-ash samples exhibited carbonaceous particles in the form of soot aggregates with primary particle size typically between 20 and 50 nm sometimes mixed or coated with multi-element inorganic species [93]. It seems possible that the soot particles were an important component in driving the adverse effects in ways analogous to the effects of diesel soot and NPCB.

There are no further studies on the ability of the NP fraction of CFA to cause oxidative stress or signal for inflammatory gene expression but such studies are warranted and we would predict that, along with the other CDNP discussed here, the pathway shown in Figure 2 would be activated, leading to inflammation.

## CDNP and the cardiovascular system

Large scale epidemiological studies suggest that inhaled ambient air pollution particles (PM_10_) may also have effects on the cardiovascular system. Small increases in particulate levels are associated with more cardiovascular deaths and hospital admissions in both time-series [94,95] and population studies [96,97]. Cohort studies have documented an association between elevated particulate and the onset of acute myocardial infarction [98,99], an increase in heart rate [100] and a decrease in heart rate variability [101]. Human chamber studies delivering concentrated ambient particles (CAPs) have confirmed that particulate can have direct effects on cardiovascular physiology with alterations in heart rate variability [101] and brachial artery diameter [102].

This CAPS work has not been able to discriminate which size fraction is responsible for any effects but the hypotheses relating to cardiovascular effects of CAPS (and PM in general) are as follows 1) particle-induced lung inflammation affects the endothelium, thrombotic potential, fibrinolytic balance and atheromatous plaque activity in ways that favour plaque rupture and thrombosis; 2) particles enter the interstitium and/or cause inflammation which affects the autonomic nerve endings that regulate the heart rhythm leading to dysrhythmia; 3) particles translocate to the blood and have direct effects on the endothelium, plaques and thrombogenic mechanism. In various models NP are shown to be highly potent in these three areas of effect i.e. NP are very potent at causing inflammation, they interstitialise readily and they can gain access to the blood. For these reasons CDNP, the principal NP in ambient air, are implicated in the cardiovascular effects of PM in these CAPS studies.

These studies address the population risks associated with ambient particulate, but do not allow any assessment of the contribution of individual air pollutants. In the Copenhagen Male Study the influence of occupational exposure on cardiovascular risk was assessed. In these men, 5 years or more of occupational exposure to welding fumes doubled the risk of myocardial infarction with exposure to solder and plastic fumes conferring similar increases in risk [103].

**Figure 3:**
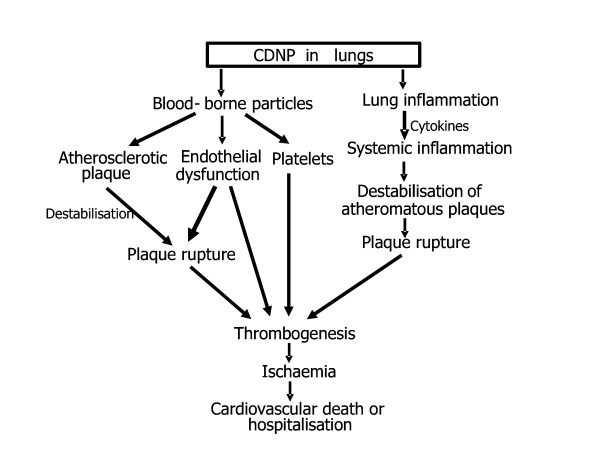


Figure 3 shows the two predominant mechanistic pathways hypothesised to mediate the adverse cardiovascular effects of CDNP [104,105]. On the right of Figure 3 inflammation caused by the CDNP is seen to affect the systemic inflammatory response and cause destabilisation of atheromatous plaques. On the left, bloodborne CDNP affect endothelial cells, platelets and plaques directly to enhance thrombogenesis.

CDNP are capable of eliciting an inflammatory response in the lung which could have stimulatory effects on leukocytes and other cells in the atherosclerotic plaques, leading to their rupture. This could occur is in the absence of any transfer of CDNP from the lungs to the circulation as it might rely on cytokines and other mediators which were released into the circulation in response to events in the lungs, affecting events in the plaques. However, CDNP could also have effects on the cardiovascular system by virtue of their ability to gain access to the bloodstream. This has been demonstrated in animal studies for a range of nanoparticles delivered by inhalation and instillation [106-111]. Once circulating, CDNP may interact with the vascular endothelium, or have direct effects on atherosclerotic plaques by entering them and causing local oxidative stress and pro-inflammatory effects similar to those caused in the lungs. Increased inflammation could destabilise the coronary plaque, resulting in rupture, thrombosis and acute coronary syndrome [104]. Furthermore, particles may interact with circulating coagulation factors to promote thrombogenesis. There is, as yet no published data demonstrating that the CDNP described here gain access to the blood in humans, but the animal studies suggest that this is a plausible hypothesis.

## CDNP and the brain

Recent work by Oberdorster and colleagues has demonstrated the transfer of radiolabelled nanoparticulate carbon from the nose of rats directly into the brain [112]. It is postulated that this transfer occurs via the olfactory nerves which run from the roof of the nasal cavity in to the olfactory lobes of the brain. However, this part of the brain is well vascularised, providing a potential systemic portal of deposition. If size is the factor that drives these effects then there is some concern that CDNP may have the general property of tropism to the brain. A search of relevant terms showed no published studies pertaining to brain transfer of diesel or coal fly-ash. The best-studied of the CDNP in regard to brain transfer is welding fume. Rats exposed to stainless steel welding fume over 60 days showed accumulation of manganese in the blood and liver but, most importantly, also in various areas of the brain [113]. Erikson et al [114] showed that inhalation exposure to manganese sulphate and manganese phosphate produced oxidative stress in the brains of rats as shown by metallothienin and glutamine synthetase levels; this varied between sexes and with age. In a sub-chronic exposure study with manganese phosphate there was accumulation of Mn in the brain but there was no associated loss of neurons or neurobehavioural effects [115]. Studies of workers exposed to welding fume, however, show clear evidence of neurological disease [116] and Mn is implicated in these effects [117]. It is not known whether the welding fume particles themselves are transferred to the brain or only the soluble Mn and other metals. However, soluble metals are very rapidly lost from welding fume particles [10] and a soluble salt of Mn was more efficient than an insoluble Mn salt in gaining access to the brain following inhalation exposure in rats [118]. Further work is required to improve our understanding of the factors dictating the transfer of CDNP and their associated soluble contaminants to the brain.

## CDNP and the liver and spleen

As discussed above, NP of various types are reported to gain access to the blood. Stuart showed in 1970 that the liver and the spleen turn black following the instillation of carbon particle into the blood because the spleen and the liver have sinusoidal phagocytes which are in contact with the blood – the 'littoral' macrophages [119]. There is existing evidence that particles can gain access to the blood since coalworkers, who receive considerable exposure to particles, show greater numbers of particles in their spleen and liver at autopsy than non-coalworkers [120]. The amount of particulate in the spleen and liver, which can be assumed to have travelled via the blood, was greater in coalworkers with more severe lung disease, suggesting that inflamed/damaged lungs may be more susceptible to egress of particles into the blood than normal lungs. The normal function of the littoral macrophages of the spleen and liver is probably to 'sample' for antigens and xenobiotics in the blood and to quickly remove any bacteria that gain access to the blood. We may therefore anticipate that any NP that gain access to the blood will be taken up by these littoral macrophages in the spleen and the liver. Particles may also reach hepatocytes and other spleen cells with consequences that are presently unknown. However, in line with the above arguments pertaining to the potential role of NP in the adverse effects of PM_10_, we may anticipate that increases in acute phase proteins during periods of high PM [121] could be due to direct particle effects on the liver, the primary source of acute phase proteins [122]. In the single study that has so far been published concerning the effects of bloodborne NP on liver function of healthy mice, CDNPs induce platelet accumulation in the hepatic microvasculature that was associated with pro-thrombotic changes on the endothelial surface of hepatic microvessels. [123]. The accumulation of particles in the liver exerted a strong pro-coagulatory effect but did not trigger an inflammatory reaction. The effects of a particle burden on the spleen are unknown but could include adjuvant effects as observed with diesel particles and antigens in the lung [124].

## CDNP and genotoxicity

The genotoxic properties of various particle types has been the focus of several studies concerned with elucidating the role such properties play in particle-associated pathogenicity [34]. However, the mechanisms involved in particle-induced genotoxicity remain poorly understood as particles are uniquely complex compared with soluble genotoxic/carcinogenic compounds, due to their physical and chemical characteristics [125]. There is evidence that 3 of the 4 CDNP studied in this review (diesel, NPCB and welding fume) are carcinogenic in humans or rats [20,38,126,127]. As mentioned earlier, DEP consist of a carbon core with adsorbed PAHs, quinones and transition metals. Genotoxicity, may therefore be caused by the direct (primary) interaction of PAHs which are known to cause DNA adduct formation [128] or alternatively via DNA strand breakage due to the production of reactive oxygen species generated by associated transition metals [8]. Carbon black particles are generally almost free of adsorbed organic compounds; however they have been shown to produce lung tumours in rats following chronic inhalation and instillation studies [127,129]. This indirect (secondary) genotoxicity pathway involves the phenomenon of lung particle overload resulting in a chronic inflammation and hence excessive ROS production leading to DNA damage. Studies by Knaapen *et al*, have demonstrated that co-incubation of rat lung epithelial cells with activated neutrophils *in vitro *stimulate the formation of the oxidative DNA lesion 8-OH-dG [32]. Less research has been carried out on the genotoxic effects of welding fumes. Some of the major components of welding fumes include iron, manganese, chromium and in particular hexavalent chromium (Cr^VI^) chromium which has been shown to increase levels of 8-OH-dG in rats after inhalation exposure [130]. Yu and co-workers showed that rats exposed for 30 days to manual metal arc stainless steel (MMA-SS) welding fumes, exhibited increased DNA damage as measured by the comet assay and immunohistochemistry for 8-OH-dG [131]. Studies investigating the genotoxic capacity of coal fly ash have shown a role for particulate size and iron release leading to radical generation and oxidative DNA damage [132,133] as well as increased sister-chromatid exchange (SCE) frequencies in peripheral blood lymphocytes from workers occupationally exposed to coal fly ash [134].

## Conclusion

**Figure 4:**
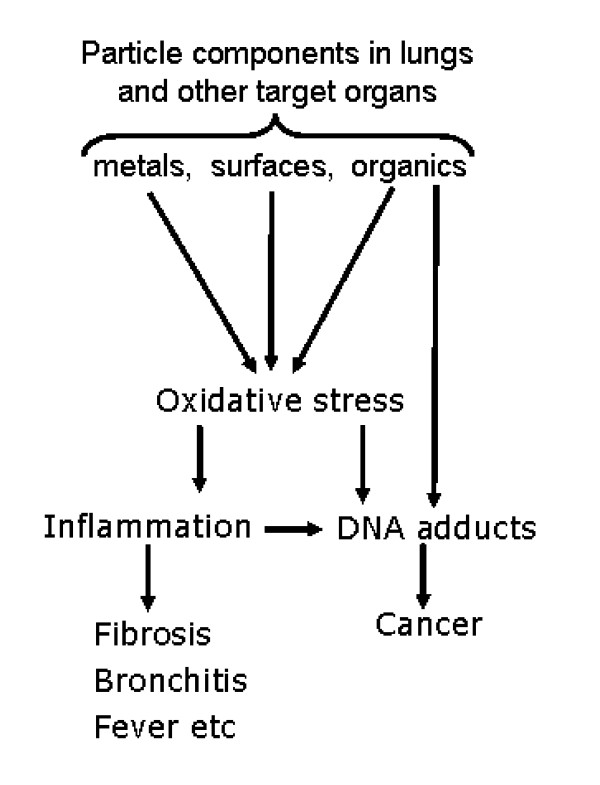


Combustion is considered a source of toxic chemicals and particles [1] and this review has focused solely on the toxicology of the particulate component. Emanating, as they do, from very diverse combustion scenarios, CDNP have received variable and piecemeal research attention. This review has used diesel soot, welding fume, carbon black and fly-ash as exemplar CDNP to demonstrate that different CDNP in fact have many properties in common that suggest that they can be viewed as a coherent class of particulate toxins. They are unified by their combustion origin, small size, universal mechanism of injury and common properties of translocation which have the potential to mediate a range of adverse effects in the lungs and other organs. Notably, the CDNP studied here all have the potential to cause oxidative stress as an integral part of their pathogenic mechanism. This oxidative stress can cause inflammation and its local and systemic acute and chronic sequelae, as well as causing oxidative adducts in epithelium that can contribute to carcinogenesis. CDNP originating from any source can therefore be considered a potential hazard to the lungs and other systems through the pathways of oxidative stress, inflammation and carcinogenesis. This is summarised in Figure 4 where the link between oxidative stress and inflammation-related effects are shown along with carcinogenic effects of oxidative stress.

Of course the temperature, conditions and substrate for combustion mean that there is considerable heterogeneity in composition between, for example, welding fume and diesel soot. Therefore the key oxidative stress event may originate from different components depending on the particle under consideration. Components that may cause oxidative stress include CDNP-associated surfaces, metals or organics; this oxidative stress then acts through oxidative stress-responsive signalling pathways to affect responses such as inflammation and proliferation. In addition, oxidative stress can also cause oxidative genotoxic DNA adducts such as 8-OH-dG whilst bulky PAH-derived adducts may also form. Both adduct types can lead to mutation. Different components of CDNP may interact to enhance the level of oxidative stress, as in the case of metals and organics interacting in the redox-cycling of quinoid organics [17] or CDNP surfaces and transition metals interacting additively in their ability to cause inflammation [81].

In addition to the local inflammatory effects of CDNP at their sites of deposition they have the potential to translocate away from their site of deposition to the blood and brain. Bloodborne particles will be delivered to the cardiovascular system, spleen and liver. The cardiovascular system has emerged as a target for the effects of PM_10_ [104, 135] and it is likely that the CDNP in PM in fact mediate this effect [3-6]. They may do this through causing inflammation in the lungs which then impacts on inflammatory processes in the atheromatous plaques that govern their stability and development. Inflammation in the lungs may also affect the thrombotic potential of the blood. Alternatively, direct effects of bloodborne particles on the endothelium, clotting system and on atheromatous plaques could be responsible. Bloodborne CDNP may deposit in the spleen, liver and heart and in these situations they may have numerous additional adverse effects.

Combustion is a ubiquitous in the modern world and the generation of CDNP is correspondingly omnipresent. Much research emphasis has been placed on traffic-derived CDNP in PM and rightly so as they are the source of most CDNP in our cities where the greatest potential for human exposure exists. However it is also clear that there is considerable potential for mixed exposures to occur in specific scenarios, e.g. a welder working in a busy street. The interactions between different particle types are unknown but the common pathway of oxidative stress means that there is potential for additive or synergistic effects. Furthermore the involvement of oxidative stress in a number of chronic diseases such as asthma, COPD and coronary artery disease argues a powerful case for existence of susceptible populations, already well-studied in the adverse effects of PM.

CDNP represent an interesting and ubiquitous category of pathogenic particles whose adverse effects are substantial and additions to the list of candidate CDNP are to be anticipated. More research is warranted into the effects of CDNP at numerous levels from factors dictating their translocation between organs and tissue to their effect in sub-cellular signalling. Viewing CDNP as a class of particles with common origins and a strong hypothesis-based understanding of their toxic mechanisms should provide impetus and direction to research on existing and new CDNP, leading to a greater understanding.

## Competing interests

The author(s) declare that they have no competing interests.

## Authors' contributions

KD planned the study and contributed to all the sections; LT contributed to all of the sections; LAJ contributed to all of the sections except the cardiovascular section; RD wrote the section ongenotoxicity and contributed to the other sections; DN and NM wrote the section on cardiovascular effects and contributed to all of the sections; WMacN contributed to all of the sections; VS contributed to the planning of the paper and contributed to all of the sections.
